# Network Analysis of Symptoms Co-Occurrence in Chronic Fatigue Syndrome

**DOI:** 10.3390/ijerph182010736

**Published:** 2021-10-13

**Authors:** Sławomir Kujawski, Joanna Słomko, Julia L. Newton, Natalie Eaton-Fitch, Donald R. Staines, Sonya Marshall-Gradisnik, Paweł Zalewski

**Affiliations:** 1 Department of Exercise Physiology and Functional Anatomy, Ludwik Rydygier Collegium Medicum in Bydgoszcz, Nicolaus Copernicus University in Torun, 85-094 Bydgoszcz, Poland; jslomko@cm.umk.pl (J.S.); p.zalewski@cm.umk.pl (P.Z.); 2 Population Health Sciences Institute, The Medical School, Newcastle University, Newcastle-upon-Tyne NE2 4HH, UK; julia.newton@newcastle.ac.uk; 3 National Centre for Neuroimmunology and Emerging Diseases, Menzies Health Institute of Queensland, Griffith University, Gold Coast, Brisbane, QLD 4222, Australia; ncned@griffith.edu.au (N.E.-F.); don.staines@griffith.edu.au (D.R.S.); s.marshall-gradisnik@griffith.edu.au (S.M.-G.); 4 Consortium Health International for Myalgic Encephalomyelitis, Menzies Health Institute Queensland, Griffith University, Gold Coast, Brisbane, QLD 4222, Australia

**Keywords:** post-exertional malaise, myalgic encephalomyelitis, epidemiology

## Abstract

Chronic fatigue syndrome (CFS) is a heterogenous disorder of multiple disabling symptoms with complex manifestations. Network analysis is a statistical and interrogative methodology to investigate the prevalence of symptoms (nodes) and their inter-dependent (inter-nodal) relationships. In the present study, we explored the co-occurrence of symptoms in a cohort of Polish CFS patients using network analysis. A total of 110 patients with CFS were examined (75 females). The mean age of the total sample was 37.93 (8.5) years old while the mean duration of symptoms in years was 4.4 (4). Post-exertional malaise (PEM) was present in 75.45% of patients, unrefreshing sleep was noted in 89.09% and impaired memory or concentration was observed in 87.27% of patients. The least prevalent symptom was tender cervical or axillary lymph nodes, noted in 34.55% of the total sample. Three of the most densely connected nodes were the total number of symptoms, sore throat and PEM. PEM was positively related with impairment in memory or concentration. Both PEM and impairment in memory or concentration presence are related to more severe fatigue measured by CFQ and FIS. PEM presence was positively related with the presence of multi-joint pain and negatively with tender lymph nodes and muscle pain. Sore throat was related with objective and subjective autonomic nervous system impairment. This study helps define symptom presentation of CFS with the pathophysiology of specific systems and links with multidisciplinary contemporary molecular pathology, including comparative MRI.

## 1. Introduction

Chronic fatigue syndrome (CFS) is a potentially severe, multisystemic illness in which patients experience a wide range of symptoms that are broadly categorised as neurological, immunological, autonomic and endocrinological [1]. Currently, there is no laboratory diagnosis available for CFS; instead, diagnosis entails the fulfilment of a diagnostic checklist. A number of different diagnostic criteria exist, and a systematic review identified 20 diagnostic criteria for CFS, emphasising the large variations in symptom heterogeneity [2]. However, there is no international standard regarding the diagnostic criteria to use and the existing criteria differ considerably by either the number of required symptoms, time of diagnosis and/or the requirement of post-exertional malaise (PEM). For instance, the number of symptoms required differs from one in the case of Oxford criteria [3], five in the Fukuda criteria [4] and eight in Canadian Consensus Criteria (CCC) [5] and International Consensus Criteria (ICC) [1].

The symptom heterogeneity of CFS patients has implications for pharmacological treatment in view of selective nature of pharmacological agents. Improvements in diagnostic methods would thus enable a more precise approach to patients’ diagnosis and treatment [6]. However, a limitation to treatment is the elusive pathomechanism underlying CFS. Following the identification of impaired ion channels, impeded immune activation and brainstem abnormalities, research has classified CFS as a neuroimmune disorder and has suggested that CFS may fall under the broad category of an ion channelopathy [7,8].

In the current investigation, network analysis was used to explore symptom co-occurrence in a cohort of CFS patients. Both subjective (fatigue, subjective autonomic nervous system functioning, symptoms from Fukuda criteria) and objective symptoms (hemodynamic and autonomic variables, body composition, symptom duration) were measured.

## 2. Materials and Methods

### 2.1. Patients

We selected 110 patients with CFS using the Fukuda criteria [4]. The inclusion criteria for the study were as follows: (1) age between 25 and 65 years, men and women; (2) fatigue more than 6 months, due to unknown causes; and (3) at least four additional symptoms: malaise after exertion, impaired memory and/or concentration, headache, unrefreshing sleep, tender lymph nodes (cervical or axillary), sore throat and muscle or joint pain. The exclusion criteria were present illness that might trigger chronic fatigue (e.g., cardiovascular disease, autoimmune disease or psychosocial causes). Patients could participate in this study if they had been referred by a general practitioner, neurologist and/or psychiatrist. The pre-test health state assessment included basic psychiatric and neurological, clinical examinations. The consultant confirmed the inclusion and exclusion criteria and verified whether an extensive physical examination and laboratory research tests had been performed to exclude any underlying illness. The mean age patients was 37.93 (8.5) and the mean duration of symptoms in years was 4.4 (4). Twenty-six patients were taking one drug or dietary supplement, sixteen were taking two and five patients were taking three chemical compounds. Twenty-three patients were taking dietary supplements (20.9%). Seven patients were taking antidepressants (6.36%).

### 2.2. Measures and Procedure

The clinical examination was performed in the chronobiology laboratory (temperature 22 °C, humidity 60%, windowless and sound-insulated room) at approximately the same time of day.

#### 2.2.1. Chronic Fatigue Syndrome Diagnosis

Symptoms from the Fukuda criteria of CFS diagnosis were measured [4]. All patients who were eventually included into analysis were characterised by chronic fatigue that persists or relapses for ≥6 months. CFS was clinically evaluated, unexplained and of new or definite onset (was not regarded as a lifelong symptom). CFS was not a consequence of chronic exertion, not decreased significantly by rest and results in substantial reduction in previous levels of occupational, educational, social or personal activities [4]. In addition, chronic fatigue was accompanied by the presence of other symptoms such as impaired memory or concentration, sore throat, tender cervical or axillary lymph nodes, muscle pain, multi-joint pain, new headaches, unrefreshing sleep and post-exertion malaise [4]. To examine the co-existence of symptoms, the criteria of a minimum number of symptoms was not applied. Symptoms were coded as “1” if present and “0” if not.

#### 2.2.2. Fatigue Measurements

The Chalder Fatigue Questionnaire (CFQ), Fatigue Severity Scale (FSS) and Fatigue Impact Scale (FIS) were used to evaluate fatigue severity. The Chalder Fatigue Questionnaire consists of 11 questions separated into two dimensions of fatigue severity: physical (items 1–7) and mental fatigue (items 8–11). This scale was scored in Likert style asking individuals to rank fatigue from 0 to 3 with a total possible range from 0 to 33. The mean Likert score was 24.4 (SD 5.8) and 14.2 (SD 4.6) [9].

The Fatigue Severity Scale (FSS) consists of nine sentences that assess the fatigue symptoms’ intensity. Questions were related to symptoms experienced in the past week, and answers were provided on a scale of 1 to 7 [10].

The Fatigue Impact Scale (FIS) consists of 40 items expressed on a 5-point scale (0–4). The total score is from 0 to 160, where a higher score refers to a greater impact [11].

#### 2.2.3. Subjective Assessment of Autonomic and Cardiovascular Functions

Participants completed the Autonomic Symptom Profile [12] as a self-report measure of autonomic symptoms. Scoring was performed using the recently abbreviated and psychometrically improved version of this questionnaire, the Composite Autonomic Symptom Score 31 (COMPASS 31) [13]. The total score as well as its subcomponents were included in the analysis (orthostatic intolerance, disturbances of vasomotor, secretomotor, gastrointestinal, bladder, pupillomotor origin).

#### 2.2.4. Objective Assessment of Autonomic and Cardiovascular Function

Hemodynamic and left ventricular function parameters were heart rate (HR), systolic blood pressure (sBP), diastolic blood pressure (dBP) and cardiac index (CI). Autonomic parameters were ratio of low-frequency (LF) diastolic blood pressure to high frequency (HF) of R to R interval (LF/HF) and left ventricular ejection time (LVET). The first 5 min were not recorded and served for to normalise the parameters. Measurements were taken in supine using Task Force Monitor (TFM, CNSystems, Medizintechnik, Graz, Austria). Beat-to-beat analysis was taken using electrocardiogram (ECG), impedance cardiography (ICG), oscillometric and non-invasive continuous blood pressure measurements (oscBP, contBP). Power spectral analysis for heart rate variability (HRV) and blood pressure variability (BPV) was conducted using the adaptive autoregressive model [14,15,16].

#### 2.2.5. Body Composition Analysis

To measure body composition changes, a multi-frequency bioelectrical impedance analyser (Tanita MC-180MA Body Composition Analyzer, Tanita UK Ltd., Manchester, UK) was applied. All subjects were attributed a “normal” proprietary algorithm for the impedance measurement.

### 2.3. Statistical Analysis

Mean and standard deviation (±SD) values are presented. The *Kendall* rank correlation coefficient was used to assess the correlation (τ and *p*-value) between the presence of particular symptoms from the Fukuda criteria with each other and their total number, as well as personal data (age, gender, length of disorder history), variables indicating subjective (Compass-31 scale and its components) and objective autonomic nervous system functioning (HR, sBP, dBP, CI, LF/HF, LVET) and fatigue scales (CFQ, FIS, FSS). The correlation between total number of symptoms and gender was examined using the *Kendall* rank correlation coefficient. Spearman’s rank correlation coefficient was used to assess the correlation (rho and *p*-value) between total number of symptoms from the Fukuda criteria with age, length of disorder history, variables indicating subjective and objective autonomic nervous system functioning and fatigue scales. The Benjamini–Hochberg adjusted *p* value was chosen to control for false discovery rate (FDR) separately for correlation between symptoms from the Fukuda criteria with each other and between Fukuda symptoms and other variables. An online calculator available at https://www.sdmproject.com/utilities/?show=FDR, access date: 30 August 2021) was used to perform a correction for the number of correlations examined. Network analysis was performed on correlations that remained significant after FDR adjustment only.

The undirected network analysis was performed using Cytoscape software version 3.8.1 [17]. The variables were grouped according to categories illustrated by the colour of the nodes: each Fukuda criteria PEM (hot pink), multi-joint pain (bisque), muscle pain (plum), new headaches (magenta), unrefreshing sleep (dark grey), impaired memory or concentration (dark blue), sore throat (pink), tender lymph nodes (auburn) and their total number (light green) are shown in the centre. Nodes indicating objective hemodynamic and autonomic parameters are shown in the cherry colour. Results of fatigue scales are shown in light blue. The total results of the Compass-31 scale (Compass Total) indicating subjective autonomic nervous system disturbances and divisions for components (secretomotor, pupillomotor, vasomotor, gastrointestinal, bladder, orthostatic intolerance) are denoted by bright red nodes. The size of the dots next to the variables names is continuously related to the number of statistically significant correlation coefficients with other variables (assuming alpha = 0.05). Statistically significant correlations after FDR correction only are presented in the form of edges. The colour of the edges denotes the direction of correlation: blue indicates negative while red shows a positive correlation. Edge width denotes the strength of the relationship. Nodes were placed in a way to increase graph visibility.

## 3. Results

In total, 110 patients with CFS were examined (75 females). The mean age of the total sample was 37.93 (8.5) years. The mean duration of symptoms in years was 4.4 (4) for the total sample, 3.69 (3.7) for females and 5.87 (4.4) for males. Body composition comparison between sexes is presented in the Table 1. Overall, 63 patients (59.43%) were taking no drugs (Table A1).

**Figure 1:**
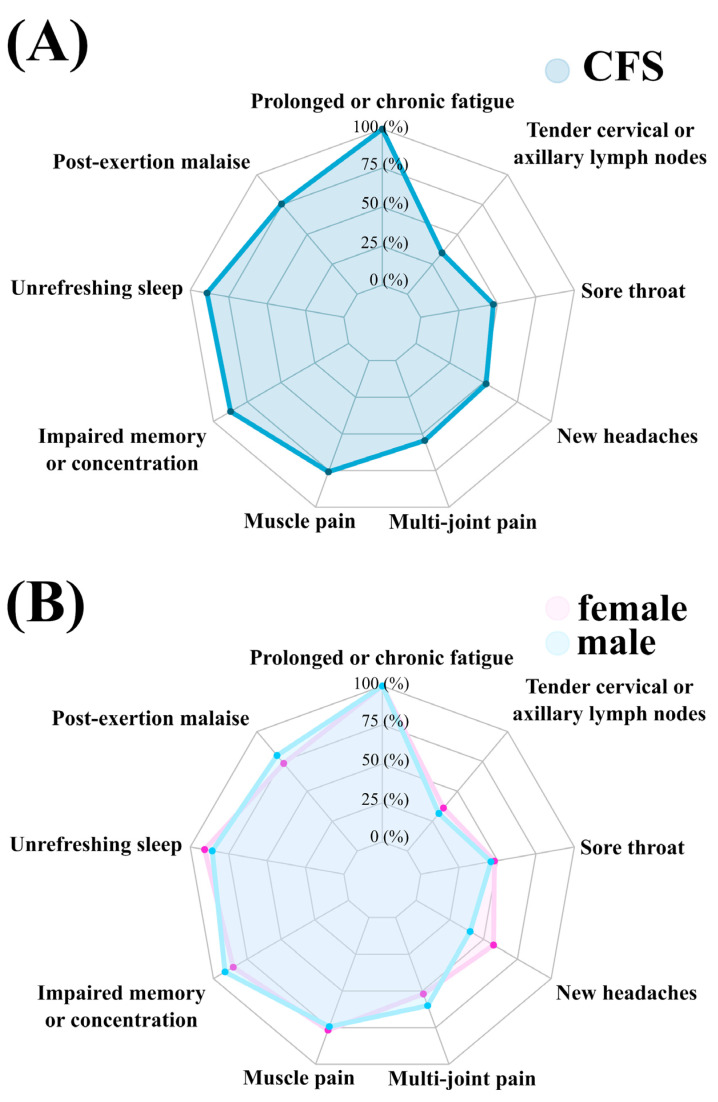
Spider chart on the prevalence of Fukuda symptoms in the whole sample (**A**) and in females and males (**B**).

Figure 1 and Table A2 present the prevalence of Fukuda prevalence in the whole sample and in females and males. All participants suffered from prolonged or chronic fatigue, a symptom which is required for CFS diagnosis according to Fukuda’s criteria. PEM, the presence of which is not required for CFS diagnosis in Fukuda’s criteria, was present in 75.45% of the total sample, 80% of males and 73.33% of females. High prevalence of reported unrefreshing sleep was noted (89.09% in total sample, 85.71% of males and 90.67% of females). In addition, high prevalence of impaired memory or concentration was observed (87.27% in total sample, 91.43% of males and 85.33% of females). The least prevalent symptom was tender cervical or axillary lymph nodes noted in 34.55% of the total sample, 31.43% of males and 36% of females. The most prominent difference between the sexes was the slightly higher prevalence of a new type of headache (40% in males vs. 57.33% in females). Overall, the mean number of total symptoms was 5.5 (1.35) per patient.

**Figure 2:**
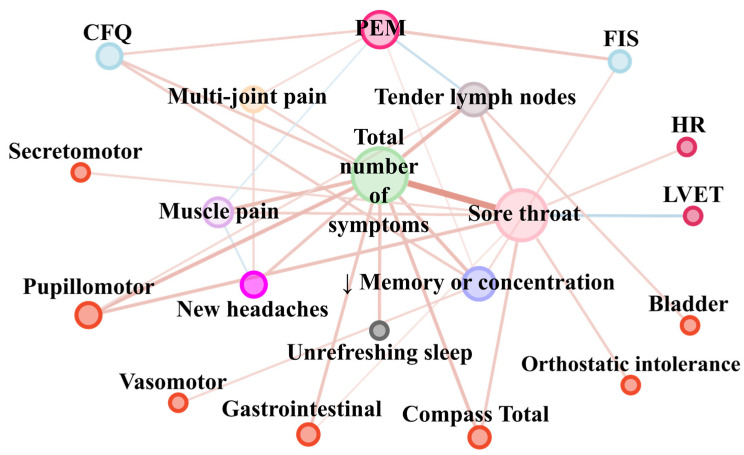
Network analysis of Fukuda symptoms’ coexistence with each other and fatigue scales, subjective and objective autonomic nervous system disturbance. Note: The variables were grouped according to categories illustrated by node colour. Each Fukuda criteria (post-exertional malaise (PEM) (hot pink), multi-joint pain (bisque), muscle pain (plum), new headaches (magenta), unrefreshing sleep (dark grey), ↓ memory or concentration (impaired memory or concentration (dark blue)), sore throat (pink), tender lymph nodes (auburn) and their total number (light green) are shown in the centre. Nodes indicating objective hemodynamic and autonomic parameters (HR—heart rate and LVET—left ventricular ejection time) are shown in cherry colour. Results of fatigue scales are shown in light blue (CFQ—Chronic Fatigue Scale, FIS—Fatigue Impact Scale). The total result of the Compass-31 scale (Compass Total), indicating subjective autonomic nervous system disturbances and divisions for components (secretomotor, pupillomotor, vasomotor, gastrointestinal, bladder, orthostatic intolerance), are denoted by bright red nodes. The size of the dots next to the variable names is continuously related to the number of statistically significant correlation coefficients with other variables. The colour of the edges denotes the trend of the correlation: blue indicates a negative correlation, while red indicates a positive correlation. Edge width and intensity of colour denote strength of relationship.

Figure 2 presents the results of correlation analysis between symptoms from Fukuda’s criteria with each other and their total number, and with the results of the fatigue scales and objective and subjective autonomic function. The matrix correlation, which Figure 2 is based on, is shown in Table 2. Three of the most densely connected nodes are total number of symptoms, sore throat and PEM. PEM was positively related with the impairment of memory or concentration (i.e., co-occurrence of both symptoms was noted) (*tau* = 0.16, *p* = 0.03). Both PEM and impairment of memory or concentration are related to more severe fatigue measured by CFQ and FIS (Figure 2, Table 2). PEM presence was positively related with the presence of multi-joint pain (*tau* = 0.2, *p* = 0.008) and negatively with tender lymph nodes (*tau* = −0.25, *p* = 0.0007) and muscle pain (*tau* = −0.17, *p* = 0.03). Sore throat coexists with the highest total number of symptoms in comparison to the rest of the Fukuda criteria symptoms (Figure 2, Table 2). In addition, sore throat was related with higher HR (*tau* = 0.23, *p* = 0.005) and orthostatic intolerance severity (*tau* = 0.27, *p* = 0.0009), and the total result of the Compass-31 (*tau* = 0.3, *p* = 0.0002) scale indicates subjective autonomic assessment and with lower LVET (*tau* = −0.3, *p* = 0.0002).

## 4. Discussion

In the current investigation, 110 patients with CFS diagnosed using Fukuda’s criteria were examined. Network analysis was used to explore symptom co-occurrence in a cohort of CFS patients.

PEM was present in 75.45% of the total sample, 80% of males and 73.33% of females, which is in accordance with previous studies. It is worth noting that PEM presence is not required for CFS diagnosis in the Fukuda criteria, yet mandatory in CCC. PEM is considered the central symptom of CFS, but it is reported as having a heterogeneous presentation in CFS cohorts [18]. There are various aspects of PEM, including but not limited to timing of onset, triggers and symptoms, that are exacerbated. In another investigation by Holtzman and colleagues, 72.3% of respondents experienced PEM immediately following exertion, while 91.4% experienced delayed onset. Interestingly, of the respondents, 78.2% reported “basic activities of daily living” to be a trigger for PEM [19], demonstrating the debilitating implications of PEM on CFS and its consequences on quality of life.

Unsurprisingly, high prevalence of reported unrefreshing sleep was noted in the above sample (87.27% of the total sample, 91.43% of males and 85.33% of females). This is in accordance with previous studies. The prevalence of sleep disturbances in CFS patients ranges from approximately 46% to 91% [20,21]. A longitudinal investigation of 65 CFS patients reported that unrefreshing sleep persisted in 79% of participants [22]. Overall, unrefreshing sleep is a common symptom in CFS and is a significant complication of PEM. For this reason, research in the area of CFS has focused on investigating non-restorative sleep and how this may contribute to illness pathophysiology [23].

The least prevalent symptom was tender cervical or axillary lymph nodes noted in 34.55% of the total sample, 31.43% of males and 36% of females. The prevalence of inflammatory symptoms ranges widely across studies [21,24,25]. In CFS, less common complaints include tender lymph nodes (37–39%), abdominal pain (32%) and sore throat (25–28%) [26]. This is consistent with the current investigation, whereby the least prevalent symptom was tender cervical or axillary lymph nodes, noted in 34.55% of the total sample, 31.43% of males and 36% of females. There is extensive evidence of immune impairment in CFS, including but not limited to references [27,28,29,30].

Surprisingly, length of CFS history was not significantly related with any symptoms after FDR correction, and therefore, this was not included in the network analysis. Three of the most densely connected nodes are total number of symptoms, sore throat and PEM. This is consistent with previous findings, wherein patients frequently report the worsening of symptoms following exertion, including cognitive exhaustion (97.4%), muscle pain (87.9%) and flu-like symptoms (86.6%) [19].

PEM was positively related with impairment of memory or concentration (i.e., co-occurrence of both symptoms was noted). Both PEM and impairment of memory or concentration presence are related to more severe fatigue measured by CFQ and FIS. Previous studies on CFS pathophysiology propose an indirect connection between PEM and cognitive dysfunction, whereby cognitive performance and brain function are significantly impacted following exertion [31]. Additionally, Chu et al. reported that difficulty of concentration and muscle pain were some of the top PEM-associated symptoms of CFS [32].

PEM was positively related with the presence of multi-joint pain and negatively with tender lymph nodes and muscle pain. Generalised pain is a common complaint reported by CFS patients. Interestingly, studies have reported that CFS patients have overall lower pain thresholds compared with healthy controls, suggesting abnormal central pain processing and impaired pain inhibition following exertion [33,34,35].

Sore throat coexisted with the highest total number of symptoms in comparison to the rest of the Fukuda criteria. In addition, sore throat was related with higher HR, orthostatic intolerance severity and total result of the Compass-31 scale, indicating subjective autonomic assessment and with lower LVET. Other investigations have reported associations have been made between immunological changes and orthostatic intolerance [36,37]. In addition, local abnormal correlations have been detected between brain magnetic resonance imaging (MRI) and peripheral blood pressure and HR [38], emphasising the communication of body systems and the role of the autonomic nervous system in CFS pathology.

The identification of symptom patterns or subgroups may be of importance for pathophysiological research. For example, this analytical approach might be helpful in identifying correlations between symptom occurrence with recently identified pathophysiology, including MRI findings and impaired ion channel function in CFS. Thus, the findings in this investigation may provide clarity in CFS heterogeneity and support the development of treatment targeted to specific node/inter-nodal connectivity resulting in symptoms.

## 5. Conclusions

PEM, unrefreshing sleep and impaired memory or concentration were three the most commonly experienced symptoms in the examined sample. Three of the most densely connected nodes were the total number of symptoms, sore throat and PEM. PEM was positively related with impairment of memory or concentration. Both PEM and impairment of memory or concentration presence are related to more severe fatigue measured by CFQ and FIS. PEM presence was positively related with the presence of multi-joint pain and negatively with tender lymph nodes and muscle pain. Sore throat was related with objective and subjective autonomic nervous system impairment. The above findings show the significance of PEM in the network of CFS symptoms. This study helps define the symptom presentation of CFS with the pathophysiology of specific systems and links with multidisciplinary contemporary molecular pathology, including comparative MRI.

## 6. Limitations and Future Perspectives

This current investigation used network analysis to explore symptom co-occurrence in CFS patients. Future investigations may employ this analytic approach in identifying correlations between symptom occurrence with pathophysiological findings. Recent publications have suggested that impaired ion channel function plays a role in the pathomechanism of CFS. Specifically, transient receptor potential melastatin 3 (TRPM3), a non-selective calcium channel, has significantly reduced expression and function in NK cells of CFS patients [7,39,40,41]. TRPM3 has a role in inflammation and nociception, which may provide a potential mechanism for the generalised pain reported by CFS patients. Moreover, TRPM3 is highly expressed in the whole brain, but limited research has commented on the function of TRPM3. Assumptions can be made concerning its function or pathophysiological role. For example, high mRNA of TRPM3 in cerebellar purkinje neurons suggests a role in coordination and movement [42]. Interestingly, neuroimaging investigations have reported abnormalities in the cerebellum of CFS patients [43,44], and CFS patients report experiencing ataxia, which is movement without coordination or loss of control of body movements [26]. Moreover, the expression of TRPM3 in the hippocampus suggests a role in memory formation and consolidation, and inflammation has been reported in the hippocampus of CFS patients [42]

This current investigation is not without limitations. Indeed, there is criticism regarding the use of the Fukuda definition for the diagnosis of CFS. However, while the Fukuda definition has been the most widely used in CFS research, more data are required to establish CFS as a neuroimmunological condition [2]. It is recommended that future research standardise the use of updated diagnostic criteria including the CCC or ICC definitions. In addition, depression should be included in the analysis. Unfortunately, in the above sample, various methods of depression assessment were used; therefore, it was eventually excluded from the analysis. In addition, objective cognitive function measurement with brain function imaging should be applied in future studies [45].

## Figures and Tables

**Table 1:** Comparison of males vs. females in body composition.

Variable	Mean (SD) Males (*n* = 35)	Mean (SD) Females (*n* = 75)
Age (years)	39.03 (8.3)	37.41 (8.6)
BMI (kg/m^2^)	25.23 (2.7)	24.14 (4.1)
FFM (kg)	64.50 (6.2)	47.74 (5.7)
Fat (%)	19.71 (4.4)	28.66 (6.7)

BMI—body mass index, FFM—free-fat mass.

**Table 2:** Symptom correlation in CFS cohort.

Pair of Parameters	Correlation Coefficient (*rho* or *tau*)	FDR *p*-Value
Bladder and Tender cervical or axillary lymph nodes	0.26	0.001
CFQ and Total number of symptoms	0.29	0.02
CFQ and PEM	0.26	0.001
CFQ and Impaired memory or concentration	0.28	0.000
Compass Total and Total number of symptoms	0.35	0.004
Compass Total and Sore throat	0.30	0.00000000006
FIS and PEM	0.31	0.0002
FIS and Impaired memory or concentration	0.21	0.01
Gastrointestinal and Total number of symptoms	0.32	0.009
Gastrointestinal and Sore throat	0.18	0.05
HR and Sore throat	0.23	0.005
Impaired memory or concentration and CFQ	0.28	0.0004
Impaired memory or concentration and FIS	0.21	0.01
Impaired memory or concentration and Vasomotor	0.23	0.005
Impaired memory or concentration and PEM	0.16	0.03
Impaired memory or concentration and Total number of symptoms	0.35	0.001
LVET and Sore throat	−0.30	0.0002
Multi-joint pain and PEM	0.20	0.008
Multi-joint pain and New headaches	0.22	0.004
Multi-joint pain and Total number of symptoms	0.25	0.03
Muscle pain and PEM	−0.17	0.03
Muscle pain and New headaches	−0.20	0.009
Muscle pain and Sore throat	0.27	0.0003
Muscle pain and Total number of symptoms	0.37	0.0007
New headaches and Muscle pain	−0.20	0.009
New headaches and Multi-joint pain	0.22	0.004
New headaches and Total number of symptoms	0.31	0.004
Orthostatic intolerance and Sore throat	0.27	0.0009
PEM and CFQ	0.26	0.001
PEM and FIS	0.31	0.0002
PEM and Impaired memory or concentration	0.16	0.03
PEM and Muscle pain	−0.17	0.03
PEM and Multi-joint pain	0.20	0.008
PEM and Tender cervical or axillary lymph nodes	−0.25	0.0007
Pupillomotor and Total number of symptoms	0.38	0.0009
Pupillomotor and Sore throat	0.34	0.00002
Pupillomotor and Tender cervical or axillary lymph nodes	0.22	0.009
Secretomotor and Sore throat	0.23	0.005
Sore throat and Orthostatic intolerance	0.27	0.0009
Sore throat and Secretomotor	0.23	0.005
Sore throat and Gastrointestinal	0.18	0.0497
Sore throat and Pupillomotor	0.34	0.00002
Sore throat and Compass Total	0.30	0.0002
Sore throat and HR	0.23	0.005
Sore throat and LVET	−0.30	0.0002
Sore throat and Muscle pain	0.27	0.0003
Sore throat and Tender cervical or axillary lymph nodes	0.31	0.00004
Sore throat and Total number of symptoms	0.61	0.00000000006
Tender cervical or axillary lymph nodes and Bladder	0.26	0.001
Tender cervical or axillary lymph nodes and Pupillomotor	0.22	0.009
Tender cervical or axillary lymph nodes and Sore throat	0.31	0.00004
Tender cervical or axillary lymph nodes and PEM	−0.25	0.0007
Tender cervical or axillary lymph nodes and Total number of symptoms	0.39	0.0003
Total number of symptoms and CFQ	0.29	0.02
Total number of symptoms and Gastrointestinal	0.32	0.009
Total number of symptoms and Pupillomotor	0.38	0.0009
Total number of symptoms and Compass Total	0.35	0.004
Total number of symptoms and unrefreshing sleep	0.32	0.004
Total number of symptoms and Impaired memory or concentration	0.35	0.001
Total number of symptoms and Muscle pain	0.37	0.0007
Total number of symptoms and Multi-joint pain	0.25	0.03
Total number of symptoms and New headaches	0.31	0.004
Total number of symptoms and Sore throat	0.61	0.00000000006
Total number of symptoms and Tender cervical or axillary lymph nodes	0.39	0.0003
unrefreshing sleep and Total number of symptoms	0.32	0.004
Vasomotor and Impaired memory or concentration	0.23	0.005

## Data Availability

Individual data are available from the corresponding author, S.K., on request.

## Appendix A

**Table A1:** Number of drugs taken by patients in the examined sample.

Group of Chemical Compound	Total Sample (*n* = 110)
	Occurrence
None	63
Diet supplements	23
Hormones	5
PPI	2
Contraception	4
Painkillers	4
Beta-blockers	1
Antiallergic	4
Other	14
Antidepresants	7

**Table A2:** Comparison of males and females in body composition.

Fukuda Criteria Symptom	Total Sample (*n* = 110)	Male (*n* = 35)	Female (*n* = 75)
	Occurrence (Percent)	Occurrence (Percent)	Occurrence (Percent)
Prolonged or chronic fatigue	110 (100)	35 (100)	75 (100)
PEM	83 (75.45)	28 (80)	55 (73.33)
Unrefreshing sleep	98 (89.09)	30 (85.71)	68 (90.67)
Impaired memory or concentration	96 (87.27)	32 (91.43)	64 (85.33)
Muscle pain	82 (75.93)	26 (74.29)	56 (76.71)
Multi-joint pain	60 (54.55)	21 (60)	39 (52)
New headaches	57 (51.82)	14 (40)	43 (57.33)
Sore throat	52 (47.27)	16 (45.71)	36 (48)
Tender cervical or axillary lymph nodes	38 (34.55)	11 (31.43)	27 (36)
